# Eating away cancer: the potential of diet and the microbiome for shaping immunotherapy outcome

**DOI:** 10.3389/fimmu.2024.1409414

**Published:** 2024-05-30

**Authors:** Ngoc-Trang Adrienne Nguyen, Yan Jiang, Jennifer L. McQuade

**Affiliations:** Department of Melanoma Medical Oncology, The University of Texas MD Anderson Cancer Center, Houston, TX, United States

**Keywords:** diet, microbiome, immunotherapy, cancer, immunity

## Abstract

The gut microbiome (GMB) plays a substantial role in human health and disease. From affecting gut barrier integrity to promoting immune cell differentiation, the GMB is capable of shaping host immunity and thus oncogenesis and anti-cancer therapeutic response, particularly with immunotherapy. Dietary patterns and components are key determinants of GMB composition, supporting the investigation of the diet-microbiome-immunity axis as a potential avenue to enhance immunotherapy response in cancer patients. As such, this review will discuss the role of the GMB and diet on anti-cancer immunity. We demonstrate that diet affects anti-cancer immunity through both GMB-independent and GMB-mediated mechanisms, and that different diet patterns mold the GMB’s functional and taxonomic composition in distinctive ways. Dietary modulation therefore shows promise as an intervention for improving cancer outcome; however, further and more extensive research in human cancer populations is needed.

## Introduction

The gut microbiome (GMB), defined as the collective genome of microorganisms inhabiting the intestinal tract, is capable of exerting significant influence in maintaining health and modulating disease susceptibility. Emerging evidence has illuminated its potential involvement in cancer development, progression, and therapeutic response (1). For instance, dysbiosis—characterized by alterations in GMB composition and function—has been associated with promotion of pro-tumorigenic environments, impairment of immune surveillance mechanisms, and increased risk and progression of certain cancers, such as colorectal cancer (1, 2). Moreover, the GMB plays a role in modulating systemic inflammation, metabolism of dietary components, and production of microbial metabolites, all of which are postulated to contribute to cancer development and progression (3). Mounting evidence also highlights the bidirectional relationship between the GMB and cancer treatment outcomes, with microbial communities potentially shaping drug metabolism, efficacy, and toxicity (2–9). Understanding the intricate interplay between the GMB and cancer thus holds immense potential for novel therapeutic strategies in cancer prevention and treatment. Patient outcomes in many cancers have improved dramatically with the approval of immune checkpoint blockade (ICB) (2–7). Immune checkpoints, such as CTLA-4 and PD-1, are proteins expressed by immune cells, and they function in promoting self-tolerance to prevent damage to healthy host tissue. However, in the context of cancer, these checkpoint pathways can inhibit anti-tumor activity by limiting T cell function and activation (10). Tumor cells can also exploit immune checkpoints by expressing partner proteins like PD-L1, further facilitating cancer immune evasion (11). ICB addresses these issues by inhibiting the binding of immune checkpoints to their partner proteins, thereby releasing limits on T lymphocyte activity and increasing recognition and destruction of cancer cells. For instance, anti-CTLA-4 (e.g., ipilimumab), anti-PD-1 (e.g., nivolumab), and anti-PD-L1 (e.g., atezolizumab) therapies are all ICB treatments that have demonstrated remarkable efficacy across various malignancies. By harnessing a patient’s own immune system and inducing immunologic memory, ICB offers the potential for long-term, durable response and, in some patients, cure. Despite this promise, clinical responses remain heterogeneous (12–16), indicating the complexity of factors influencing treatment outcomes, and actionable strategies to enhance ICB efficacy are urgently needed. Prior investigations have predominantly focused on tumor signaling pathways and the role of the tumor microenvironment (TME) in shaping ICB response. However, there is growing recognition of the pivotal role of host-related factors, including modifiable ones, in shaping systemic and anti-tumor immunity. Diet is one such factor, with recent research contributing to its emergence as an intriguing potential modulator of ICB response.

As well-reviewed in other literature, there are multiple mechanisms through which diet has been shown to impact carcinogenesis as well as mucosal and systemic immunity in both physiologic and pathogenic states (17–20). There is now emerging preclinical and observational evidence that diet may also impact anti-tumor immunity, particularly in the context of immunotherapy. Of particular interest is the interaction between diet, the GMB, and immunity, a well-defined interplay that has supported biological plausibility of how diet could play a major role in shaping immunity—especially as it is now well-established that the GMB can affect response to ICB in cancers (2–7).

Diet is a key determinant of GMB structure. The metabolic output of the GMB (i.e., its function) is also strongly shaped by diet, and is a key mechanism underlying the GMB’s influence on immunotherapy efficacy. There is thus a strong rationale for examining the diet-microbiome-immunity axis as a potential approach to improve immunotherapy response. Understanding of interactions between diet, the microbiome, and immunity is gaining clarity among other populations and settings (21–23), but this was previously largely unexplored in the setting of cancer outcomes and in ICB treatment specifically. In this review, we will discuss the impact of overall dietary patterns (e.g., Mediterranean diet), specific dietary regimens (e.g., ketogenic diet and fasting-mimicking diets), and dietary components (e.g., fiber) on the GMB and immunity. While this review is anchored in the diet/GMB/anti-tumor immunity interaction, it should be noted that diet is complex, and that nutrition could shape immune response via both GMB-dependent and GMB-independent mechanisms. For example, even within the context of dietary patterns that can shape the GMB, such as a plant-based diet vs. Western diet, immune-modulating metabolites can be host-, nutrient-, and/or GMB-derived.

The potential for diet as a low-cost, low-risk strategy to improve outcomes is appealing to both patients and physicians. Much work remains to be done to further establish causality, mechanism, and to refine rational therapeutic strategies. In this review, we will summarize the emerging evidence, current gaps, and future directions in our understanding of diet and anti-tumor immunity, with a central focus on the microbiome.

## GMB and ICB response

Given the proximity of the GMB to gut-associated lymphoid tissue, which constitutes the largest component of the human immune system, it is no surprise that the GMB has the potential to heavily influence immune (24). The GMB is crucial for maintaining gut mucosal barrier integrity and homeostasis, and shapes both innate and adaptive immune response at local and systemic levels (25). The bidirectional interplay between the GMB and immunity has been well-studied in the context of gastrointestinal and autoimmune disorders. More recently, the GMB has also been implicated in response to cancer therapies, most notably ICB (2–4, 8, 9, 26, 27). First studied in mice, the diversity, composition, and function of the GMB have now also been associated with response to ICB in humans. Importantly, the GMB is not only a biomarker of response but also a potential therapeutic target.

Transplantation of favorable/pro-ICB-response microbiota from either mice or ICB-responding patients into mouse models can enhance response to ICB, supporting a causal link between the microbiome and immunotherapy response (2–4, 8). More recently, two early phase clinical trials of FMT + anti-PD1 in anti-PD1 refractory melanoma have demonstrated that microbiome modulation in this setting is feasible, safe, and a promising approach to overcome immunotherapy resistance (27, 28), though larger studies powered for disease outcome are needed. However, there are many challenges with scaling FMT as a therapeutic approach, including availability, procedural risks, and donor selection and recruitment (29, 30). Thus, investigation of other methods to modulate the microbiome are urgently needed. Of particular relevance is a need for further investigation into diet-microbiome-immunity axis in the context of immunotherapy as a scalable, low risk approach to microbiome modulation.

There are various possible mechanisms through which the GMB can modulate the immune system and consequently immunotherapy efficacy, many of which are potentially modifiable by diet (31). In preclinical studies, favorable GMBs have been shown to promote dendritic cell (DC) and CD8+ T cell maturation, function, and response; increase frequency of activated T cells; induce cytokine secretion, and decrease peripheral T regulatory cells (Tregs) (3, 4, 8, 9). As to how the GMB induces these immune effects, there are multiple mechanisms. First, microbial activation of pattern-recognition receptors via pathogen-associated molecular patterns like lipopolysaccharides (LPS) and peptidoglycan, can alter the maturation and priming of immune cells such as DCs and T cells (32). Interestingly, though the emphasis has been on responder microbial signatures, there are also non-responder phenotypes shared across cohorts such as LPS-expressing gram-negative bacteria, which induce innate inflammatory pathways via TLR4 (6). As such, the reduction of potentially deleterious taxa may be as important a goal as enhancing beneficial bacteria, and dietary changes like reducing animal fat intake may be an avenue to accomplish this (23, 33–35). Another putative mechanism of GMB-induced immunomodulation includes molecular mimicry, wherein microbial antigens trigger antigen-specific immune responses due to resembling other antigens (31, 36). Finally, metabolites produced by the GMB, including byproducts of the GMB’s role in harvesting host-consumed nutrients, can have effects on both mucosal and systemic immunity (31, 36).

### Microbial metabolites

Microbial-derived metabolism is intimately connected with our own dietary intake. For example, riboflavin (vitamin B2) —found in dairy, meats, cereals and grains, nuts, and green vegetables (37, 38) —is metabolized by the GMB to produce derivatives that can promote expansion of mucosal-associated invariant T cells, which are seen to be activated to a greater extent in responders to PD-1 ICB (39). However, while several microbial metabolites have been linked to improvements in immunity and immunotherapy response, the same metabolites are often also implicated in cancer formation. For instance, microbial metabolism of primary bile acids from the consumption of animal fat/proteins produces secondary bile acids (SBAs), which have been implicated in hepatocellular carcinoma carcinogenesis. Despite this, the SBA ursodeoxycholic acid has been found to reduce cancer immunosuppression by inducing TGF-β degradation (40).

The role of microbial tryptophan metabolism is also controversial, with some studies suggesting indoles can act as aryl hydrocarbon receptor (AhR) agonists, promoting immune evasion in the tumor microenvironment (TME), and others showing these metabolites can promote anti-tumor immunity (41–44). One study observed that selective antibiotic targeting of *Lactobacilli*, which are indole-producers, reduced pancreatic tumor growth and increased T cell infiltration. Conversely, provision of *L. reuteri* resulted in increased tumor growth and reduction in intratumoral CD8^+^ T cells (45). On the other hand, other studies have found that microbial tryptophan metabolites can promote chemotherapy and ICB efficacy (43, 44). Recently, Bender et al. demonstrated that *L. reuteri* was able to translocate from the intestine to the tumor bed and produce indole-3-aldehyde *in situ* which *promoted* antitumor immunity and ICB response in an AhR-dependent, Tc1-cell-inducing manner (43, 44). Notably, cruciferous vegetables like broccoli, cabbage, and brussels sprouts possess dietary indole compound precursors that can activate AhR yet demonstrate reduced cancer risk in a potentially dose- and context-dependent manner (46–51).

However, the GMB metabolites that have garnered the most attention as potential mediators of ICB response are short chain fatty acids (SCFAs), which will be discussed in detail below. Many of the bacteria associated with ICB efficacy and response across studies are involved in fiber fermentation and the production of SFCAs and/or support other fiber-fermenting bacteria (2, 3, 6, 7, 52, 53). Ultimately, further investigation into the function and metabolic output of the GMB rather than its taxonomical constituents may overcome some of the lack of concordance observed across studies of microbiome and ICB response given functional redundancy of the GMB (6, 7).

## Diet, the GMB, and ICB response

Environment plays a much larger role than genetics in determining GMB composition (33, 54). One of the most important potentially modifiable factors that shapes the GMB is habitual diet, which may underlie many of the regional differences observed in GMB profiles as well as the lack of concordance in defining pro-ICB response associated taxa around the globe (6, 7, 54). Multiple pre-clinical and interventional trials have reported that specific nutrients and diet components can impact the GMB (6, 55–58). However, individuals consume foods rather than isolated nutrients, and the contribution of foods to the post-digestion substrate received by the GMB may be far more variable than the nutrient content suggests—thus, examining the impact of dietary *patterns* on the GMB, rather than the impact of specific macro/micronutrients, may provide a better understanding of the mechanisms of diet-associated health benefits and risk.

Healthy dietary patterns emphasizing plant-based foods support favorable GMB profiles with a higher abundance of species capable of carbohydrate fermentation and an increase in SCFA producers, which in turn have been linked to improved host metabolic profile and lower cancer risk (59). In addition to stimulating production of favorable metabolites, dietary fiber can also influence cancer processes by binding with and/or absorbing carcinogenic bile acids (60, 61). Conversely, “unhealthy” diets, such as those enriched in red meat, saturated fats, processed carbohydrates, added sugars, and lower fiber, may promote the outgrowth of inflammatory bacteria implicated in carcinogenesis and encourage microbial expression of genes encoding pro-inflammatory cytokines and transcription factors associated with worse cancer outcome, including with ICB (7, 62).

Diets high in animal protein are associated with higher abundance of harmful *Bacteroides* spp. and lower abundance of beneficial species like *E. rectale* and microbes in the *Lactobacillus* and *Roseburia* genera (33). Consumption of red and/or processed meat also introduces carnitine and choline into the GMB, whose eventual metabolic product trimethylamine N-oxide (TMAO) has been linked to inflammation, type II diabetes, and obesity (63, 64). However, it should be noted that TMAO abundance has been found to be associated with improved immunotherapy response in triple-negative breast cancer, indicating a need for deeper exploration of TMAO’s oncologic impact (65). Consumption of excess animal and/or saturated fats also increases LPS that promote a colonocyte pro-inflammatory gene signature (7, 23, 34, 35, 66, 67). However, high-fat diet patterns should be distinguished from ketogenic diets, which may have very different effects on the GMB and immunity as discussed in the corresponding section.

Significant intake of ultra-processed foods (UPFs) has also been associated with GMB disruptions and increased cancer risk. Made from substances extracted or derived from whole foods, UPFs are typically high in sugar, unhealthy fats, and salt, but low in protein, fiber, vitamins, and minerals. UPFs usually also contain artificial substances like sweeteners, emulsifiers, and preservatives (68, 69). The low vitamin, mineral, and fiber content of UPFs have been shown to decrease GMB diversity, richness, abundance of fiber-fermenting bacteria, and SCFA production (70–74). Moreover, emulsifiers found in UPFs, such carboxymethyl cellulose (CMC) and polysorbate 80 (P80), may promote bacterial translocation across the mucosal barrier, increase LPS synthesis, and enhance colon carcinogenesis (75–77). Observational studies have also found correlations between high UPF intake and overall cancer risk, as well as risk of specific conditions like breast, colorectal, and pancreatic cancer. Thus, while the direct, negative impact of UPFs on immunity has yet to be decisively proven, their consumption has been shown to be associated with dysbiosis and metabolome perturbations, which may thereby affect anti-cancer immunity.

While habitual diet is a major determinant of the microbiome, an important question is whether diet change can change the microbiome, i.e., whether it can be used therapeutically. In a landmark study by David et al, participants consumed one of two diets *ad libitum* over the course of 5 days: an animal-based diet, which consisted of meats, eggs, and cheeses, or a plant-based diet, which was based on grains, legumes, fruits, and vegetables (78). After the study, those in the animal-based diet arm were found to have increased abundance of bile-tolerant microbes, potentially increasing the carcinogenic burden of compounds like SBAs. The animal-based diet also increased bacterial gene expression for microbial bile salt hydrolases, which are prerequisites for the production of deoxycholic acid by the GMB, a potentially carcinogenic metabolite. In contrast, participants who consumed a plant-based diet had higher abundance of beneficial fiber-fermenting Firmicutes bacteria, such as *Roseburia* spp., *E. rectale*, and *R. bromii* (78).

Similar to the study by David et al, O’Keefe et al. performed a 2-week diet exchange between African-Americans and rural Africans. The African-American diet generally consisted of high fat, high animal protein, and low fiber content, while the rural African diet was plant-based, low fat, and high fiber content. Upon switching to the African-American diet, rural African participants displayed increased mucosal epithelial proliferation rates and greater inflammatory markers in tissue (e.g. CD68+ lamina propria macrophages). Increased SBA synthesis was also observed, with fecal SBAs increasing by almost 400% in African participants after consuming the African-American diet. Meanwhile, African-American participants consuming the rural African diet exhibited an almost 70% decrease in fecal SBAs, lower colonic mucosal inflammation and proliferation rates, and increased saccharolytic fermentation and butyrogenesis. Study participants’ reciprocal changes in mucosal/gut metabolome cancer risk biomarkers as a result of the diet swap suggest that the diet-microbiome interaction may play a key role in the higher rates of colorectal cancer found in industrialized countries (79).

### Fiber, SCFAs, and immunity

A common feature shared by plant-based diets is the possession of high amounts of dietary fiber, which is defined as nondigestible carbohydrates and lignin that are intrinsic and intact in plants (80–82). In preclinical models, fiber deprivation lowers microbial diversity (83) and decreases the abundance of beneficial fiber-fermenting bacteria such as *F. prausnitzii* (bacteria associated with response to ICB). Insufficient fiber can also cause some bacterial taxa to begin degrading host mucin glycans, thus thinning the mucus layer; promoting inflammation, bacterial translocation, and oncogenic signaling pathways; and inducing greater tumor development as a result (83, 84).

SCFAs, i.e., butyrate, acetate, and propionate, are the product of dietary fiber fermentation by gut microbiota and a major mediator of the effects of plant-based diets. SCFAs serve as the main energy source for intestinal epithelial cells (IECs) (85), ensuring mucosal barrier integrity and preventing bacterial translocation (86–89), and also have both indirect and direct effects on immunity. *In vitro* administration of SCFAs to IECs activates G protein coupled receptors (GPCRs), increases the production of anti-inflammatory cytokines like IL-10 (85), and can also abrogate neutrophil recruitment, regulating the production of inflammatory mediators like TNF-a and IL-17 and thus playing an important role in recruiting leukocytes and activating T lymphocytes (88). SCFAs can also induce plasma cell production of IgA, blocking virulence and bacterial adherence to epithelial cells (90, 91). Perhaps most relevant for ICB response is that SCFAs can also enhance T cell activity. For example, acetate has been shown to enhance IFN-γ production from CD8+ and intratumoral cytotoxic T cells (CTLs) via the mammalian target of rapamycin pathway (92) as well as memory CD8+ T cell responses via GAPDH enzyme activity and glycolysis (93). Meanwhile, the role of the SCFA butyrate in suppressing carcinogenic pathways, such as the *Wnt* signaling pathway, has been well-studied in colonocytes (94, 95). More recent studies have demonstrated that butyrate can also impact immune cell function via histone deacetylation inhibition (88), which upregulates IFN-γ and granzyme B expression while suppressing IL-17A production in CTLs, thus facilitating an increase in the switch of Tc17 cells to a CTL phenotype (92).

However, although multiple studies have demonstrated the potential benefits of fiber and SCFAs for cancer immunity, there is also evidence for their negative impact. For instance, one study found that incorporating the fiber inulin into mice’s diets induced hepatocellular carcinoma via a microbiota-dependent mechanism (96). Another experiment detected an increase in mouse colonic tumor load when the amount of fiber—consisting of either inulin, cellulose, or brewers spent grain—was increased in their diets (97). Other studies, both human and preclinical, have also found an association between fiber/SCFAs and aggravation of factors promoting cancer risk/development, such as greater production of cancer-associated cytokines (88, 98–101). These seemingly discrepant results may be driven by the specific sources of dietary fiber incorporated, types of fiber, and differences in study populations, including cancer type (102). Isolated fiber may also have different effects than whole-food-derived fiber, i.e., it is possible that the combination of fiber *and* the micronutrients found in whole-food/natural dietary fiber sources is required in order for patients to reap the potential cancer-inhibiting benefits of a high-fiber diet. Furthermore, specific types of fiber may elicit differential biological effects (97, 103, 104), with one murine study observing a significantly lower tumor load in inulin-fed mice than in mice fed cellulose or brewers spent grain (97). It thus remains to be seen how we may optimize fiber-related dietary interventions targeted at improving cancer immunity in a microbiota-dependent manner. However, given the enormous amount of data supporting the health benefits of a whole-food, high-fiber diet and the context-dependent factors that may have led to the negative effects observed in other studies, it is reasonable to recommend increasing fiber consumption through natural sources while continuing to research its impact on cancer immunity.

Given the existing literature on the immunomodulatory role of SCFAs and the known role in fiber fermentation of many pro-ICB-response bacteria, there was a strong rationale to examine the potential link between dietary fiber consumption and immunotherapy response. Indeed, a 2021 study by Spencer et al. incorporated dietary screener questionnaires into their observational microbiome cohort and found that habitual higher dietary fiber consumption was associated with significantly improved progression-free survival in melanoma patients undergoing ICB (105). Similarly, a 2022 multicenter cohort study observed that a Mediterranean diet pattern—which features high intake of plant-based fiber alongside omega-3 fatty acids and various micronutrients—was positively associated with ICB response, progression-free survival, and less immunotoxicity among patients with advanced melanoma (106). As expected, dietary fiber intake is positively correlated with pro-ICB-response, fiber-fermenting bacteria (58). These findings were recapitulated in murine melanoma models, where high-fiber diet fed mice displayed improved treatment response to anti-PD-1 ICB—with increased CD4+ T cell infiltration in tumors, and greater expression of genes linked to T-cell activation and interferon response in CD45+ TILs —compared to anti-PD1 low-fiber fed mice (105).

Rapid shifts in the GMB and lower stool concentrations of the SCFA propionate are also observed with fiber deprivation in preclinical models. Importantly, synergy between ICB and dietary fiber were not seen in germ-free mice, supporting that the effects were indeed microbiome-mediated. In another preclinical study, a high-fiber diet modulated the GMB in a manner that triggered this intratumoral IFN-1/natural killer cell (NKC)/DC axis, improving ICB efficacy and programming TME mononuclear phagocytes toward immunostimulatory phenotypes (107). Given these observational and preclinical data supporting the role of fiber in modulating the microbiome and immunity, human interventional studies (NCT03950635 and NCT04645680) are now ongoing that investigate high-fiber diet as a GMB-focused strategy to improve anti-cancer immunity. Be that as it may, future microbiome-centered dietary interventions will likely require tailoring to individual patients. Recent findings suggest that GMB compositions associated with high fiber intake—namely, high-diversity GMBs with high relative *Ruminococcaceae*, butryogen, and methanogen abundance—are associated with ICB response, but that modulating taxa composition alone may not be enough to improve outcome. For instance, having higher relative abundance of merely one putatively beneficial taxon, such as *F. prausnitzii*, may be insufficient to induce response, and was in fact observed to pose no further advantage for ICB outcome in patients already possessing high-fiber-associated GMBs prior to ICB (58). Employing high-fiber diets in the context of immunotherapy may thus be an intervention better suited for patients with a GMB composition shaped by habitual low-fiber intake [i.e., low-diversity, *Bacteroides*-dominated microbiomes (58)], but even then, tumor-intrinsic, systemic, and other factors also need to be considered.

### Dietary probiotics

Probiotics are live microorganisms with putative health benefits when consumed. They are found in fermented foods such as kimchi, kefir, sauerkraut, etc., which are “produced through controlled microbial growth … and the conversion of food components through enzymatic action” (108). As such, probiotic foods contain both live microorganisms and food sources for said microorganisms, such as non-digestible carbohydrates like inulin-type fructans and galacto-oligosaccharides (109, 110). This allows probiotic foods to support a beneficial GMB composition by encouraging the growth of bacteria like *F. prausnitzii* while pulling resources from and thus suppressing the growth of pathogenic bacteria.

These naturally occurring dietary probiotics, however, stand in contrast to commercial probiotic supplements, which are generally not targeted/rationally designed, but instead frequently composed of only aerobic, easy-to-culture bacteria in order to minimize production costs (111, 112). Commercial probiotics also lack the beneficial nutrients inherent to probiotic foods, such as essential amino acids, polyphenols, different types of fibers, and other fermentable bioactive compounds (113, 114). In fact, over-the-counter probiotic supplements have been shown to lower overall GMB diversity and worsen ICB response in preclinical models and were also associated with worse outcomes with ICB in an observational study (105). The negative impacts of unselected probiotics on GMB homeostasis has also been demonstrated after antibiotic use, wherein probiotics inhibited the restoration of normal gut ecology after antibiotics (105, 111). Thus, the use of probiotics outside the context of a clinical trial should be discouraged at this time.

Conversely, observational data suggests a beneficial impact of dietary probiotics on the GMB and immunity. Natural yogurt consumers have been found to display healthier metabolic profiles, with lower levels of inflammation and serum lipid peroxidation and higher fecal concentrations of *Akkermansia* (115, 116). In a preclinical study by Woo et al., mice with DSS-induced colitis were fed with a fermented barley and soybean mixture which led to increased intestinal barrier function and decreased levels of inflammatory cytokines in colonic tissue. The fermented mixture was also found to suppress bacterial translocation to mLNs and promote growth of the anti-inflammatory bacterial genera *Lactobacilli* and *Bacteroides*, the latter of which has been demonstrated to be an important contributor to anti-CTLA-4 ICB efficacy (9, 117).

A recent human interventional study substantiated these health-promoting effects of dietary probiotics. Wastyk et al. conducted a high-fermented-food diet intervention in healthy individuals targeting consumption of 6 servings of fermented foods daily. Post-intervention, participants exhibited increased GMB diversity; importantly, beneficial taxa that were enriched were in large part not derived from the microbes found in the fermented foods consumed, suggesting that dietary probiotics can influence the GMB composition both directly and indirectly and seem to support the overall ecosystem. They further examined the effects of this diet on circulating immunocytes, finding increased effector memory CD4+ T cells with decreased non-classical monocytes and circulating inflammatory cytokines in the post-intervention specimens (21).

### Ketogenic diets

Ketogenic Diets (KD), characterized by low carbohydrate and high fat content, aim to reduce carbohydrate intake to the point of ketosis, where ketone bodies are produced and metabolized as an energy source rather than glucose, the usual main source of energy. The premise of using the ketogenic diet in cancer is based on the “Warburg effect”, an observation that cancer cells rely primarily on aerobic glycolysis rather than mitochondrial oxidative phosphorylation (118). However, it has subsequently been shown that cancer cells are much more metabolically plastic than originally thought (119). Nonetheless, the ketogenic diet has been shown to limit cancer growth in many preclinical models via lowering of insulin and IGF-1, and intriguingly has shown synergy in combination with cancer therapies that disrupt the insulin/IGF-1/PI3K axis in host organs (120).

Recent studies have examined the impact of the KD on anti-tumor immunity and on the GMB. In murine models, Ferrere et al. found that KD inhibited tumor growth and cancer progression and promoted ICB efficacy, at least in part via the ketone body 3-hydroxybutyrate (3HB) in a T-cell dependent manner (121). Notably, systemic administration of 3HB mimicked the antitumor effects of KD, enhancing ICB efficacy and promoting CXCR3+ T cell expansion. In terms of the effects on the microbiome, KD led to an increased abundance of *A. muciniphila* and other immunogenic bacteria. The tumor-suppressive effects of KD and 3HB were attenuated by antibiotics, indicating that the microbiome may mediate the KD’s anti-cancer effects (121). Other murine studies have similarly found that KD can increase potentially beneficial bacteria like *A. muciniphila* and *Lactobacillus* while decreasing putative inflammatory taxa, such as *Desulfovibrio* and *Turicibacter* (122).

However, implementation of KD in humans, especially cancer patients, requires close supervision by registered dietitians, as hypoglycemia, weight loss, and electrolyte imbalances may occur (123). Further, KD limits fruit, vegetables, whole grains, and fiber ingestion, and thus the effects of this diet on the human microbiome need to be carefully considered. Even so, clinical data has shown some promise. In a human diet intervention study by Link et al, participants sequentially consumed KD and vegan diets for 2 weeks each and in randomized order. Both KD and vegan diet induced differential changes on host immunity and GMB composition: the KD increased activated T_reg_ and CD16^+^ NKC abundance as well as T cell activation; meanwhile, the vegan diet increased activated T helper and NKCs and upregulated innate-immunity-associated pathways (124). Further investigation is needed to elucidate the potential implications for anti-cancer immunity.

### Calorie restriction/fasting-mimicking diet

Calorie restriction (CR) is defined as consuming 50-90% of usual ad libitum calorie intake without going to the extent of malnutrition. On the other hand, a fasting-mimicking-diet (FMD) reduces calorie intake to a lesser extent and is specifically low in protein and sugar yet high in unsaturated fat content (125–129). Multiple preclinical studies have demonstrated that CR and/or fasting-like conditions reduce cancer risk, incidence, progression, and mortality, though data in humans remains limited (107, 130–133). Like the KD, a major target of CR and FMD is insulin and IGF-1 levels. High serum IGF-1 has been associated with increased risk of multiple cancers (134). In rodents, long-term CR reduces serum IGF-1 concentration by 30-40%, and fasting in humans markedly decreases serum IGF-1 concentration provided that protein intake is not excessively high (135). In terms of immune effects, preclinical studies have found that CR alters NKC maturation and function; meanwhile, fasting-like conditions have been shown in pre-clinical models to increase cytotoxic CD8 T cell frequency and reduce T_reg_ tumors in the TME while also impacting NKC maturation (107, 136–138).

FMD has been shown to enhance chemotherapy efficacy in murine breast cancer models by promoting T-cell-mediated tumor immunogenicity and cytotoxicity (107, 136–138). More specifically, FMD administration induced greater cytotoxic CD8+ T cell recruitment, greater levels of common lymphoid progenitor cells and circulating/tumor-infiltrating CD8+ lymphocytes, and decreased activation and frequency of T_reg_ cells. These effects were mediated at least in part by a decrease in expression of the heme oxygenase-1 (HO-1) enzyme in cancer cells. This downregulation of HO-1 expression served to selectively sensitize cancer cells to chemotherapy while bypassing normal cells, whose HO-1 expression levels were not impacted (137, 139, 140).

Similarly, preclinical studies have shown that FMD elevates IFN-γ levels, increases CTLs and NKCs, promotes switching of CD8+ T cells to an activated/memory phenotype, and decreases immunosuppressive myeloid-derived suppressor cells (MDSCs) and Tregs (107). A periodic FMD has also been shown sensitize cancer cells to ICB, increasing anti-PD-L1 and anti-OX40 ICB efficacy against triple-negative breast tumors. In these murine subjects, FMD cycles were found to reactivate and expand early exhausted effector T cells and induce cancer cells to switch from respiratory to glycolytic metabolism (141). Meanwhile, human studies found that fasted cancer patients who adhered to a 5-day FMD (700 kcal consumed on day 1; 300 kcal consumed days 2-5) every 21-28 days displayed greater amounts of activated T cells and cytolytic NKCs in peripheral blood (142). In another study of heterogeneously treated (not ICB) cancer patients, FMD augmented intratumoral Th1/cytotoxic responses and reduced immunosuppressive MDSCs and Tregs. The study also noted greater amounts of CTLs and NKCs in patient blood; increased intratumoral populations of CD8+ T cells, DCs, NKCs, and effector memory T lymphocytes; a heightened switch of CD8+ cells toward a memory/activated phenotype; and increased levels of T cell stimulating cytokines (107). Another clinical trial observed that administration of hydroxycitrate, a CR mimetic, lead to depletion of Tregs and thus heightened anticancer immunosurveillance and decreased tumor burden (143).

In terms of the GMB, murine studies on CR/FMD have observed an increase in populations of putative protective/beneficial bacteria, such as SCFA-producing *Bifidobacterium* and *Faecalibaculum* as well as immunogenic *A. muciniphila* (144–147). In the same vein, preclinical models have noted a decrease in reputed inflammatory taxa, such as *Helicobacter, Streptococcaceae*, and *Desulfovibrionaceae* (144, 145, 148). Therefore, FMD and CR lead to marked systemic changes in the GMB, metabolism, and immunity in ways that substantiate the further investigation of FMD/CR. However, these types of diet interventions in patients with cancer should only be utilized under physician supervision and in the context of a clinical trial.

## Conclusion

There is now a substantial body of evidence demonstrating that the GMB can impact both cancer risk and development as well as ICB response. However, mechanisms as well as the most effective and scalable strategies to modulate the microbiome to improve ICB outcomes remain to be active areas of investigation. Diet is a key determinant of the GMB and can impact immunity via both GMB-mediated mechanisms as well as GMB-independent pathways (78, 149).

Cohort studies support that a habitual high dietary fiber intake is associated with improved response to ICB (58, 105, 150), and that in preclinical models, dietary fiber manipulation can modulate ICB response via the GMB. However, it is unclear in humans whether the epidemiological association between dietary fiber and ICB response is truly from fiber specifically vs. from other beneficial nutrients found in a fiber-rich diet. Meanwhile, although diet patterns such as a plant-based vs. Western diet are captured in epidemiological dietary assessments, more “radical” diets such as the ketogenic diet and calorie restriction cannot be, yet they are much easier to study in rodent models. Thus, while there is ample literature supporting the anti-cancer effects of these diets in preclinical models, human data in cancer populations remain limited.

While diet is a relatively inexpensive, low-risk, scalable approach to potentially modulate the microbiome and improve ICB response, there are also several challenges. The exact mechanisms by which “beneficial” vs. “detrimental” dietary interventions produce their respective effects on cancer/immunotherapy need to be further defined (29). It also remains unclear if such effects are divided by tumor type: studies are limited by what model was used, and so it cannot be said with certainty that any observed effects of diet on immunity are tumor-specific. Therefore, until evidence emerges that a dietary intervention induces anti-tumor immunity in one cancer type but not another, future research should endeavor to study a diet’s effects across tumor models and patient populations to determine what effects are tumor-specific vs. agnostic. Another key question is whether the focus should be on specific nutrients/food components or on larger dietary patterns. For specific food components such as fiber, the “dose” needed to induce GMB changes has not been defined. Patient selection for microbiome-directed dietary interventions is another key question—should this be based on microbiome profiling, diet assessment, or a combination of the two? Then there is the heterogeneity and complexity of the GMB: the host factors and exposures that shape this ecosystem include ethnicity, body composition, metabolic phenotype, comorbidities, medication use, and still other lifestyle factors. In terms of timing, does the microbiome need to be primed prior to ICB initiation, and/or do diet changes need to be sustained throughout treatment? To begin addressing these questions, what is needed are rigorously and rationally designed interventional human studies with microbiome and disease endpoints. However, in addition to these questions regarding mechanism and mode, there is the additional challenge that behavior change is difficult, and supporting patients to make these changes will require provider education, increased access to dietitians or health coaches with a focus on diet optimization, tools such as apps and recipes, and ultimately public health measures.

**Figure 1:**
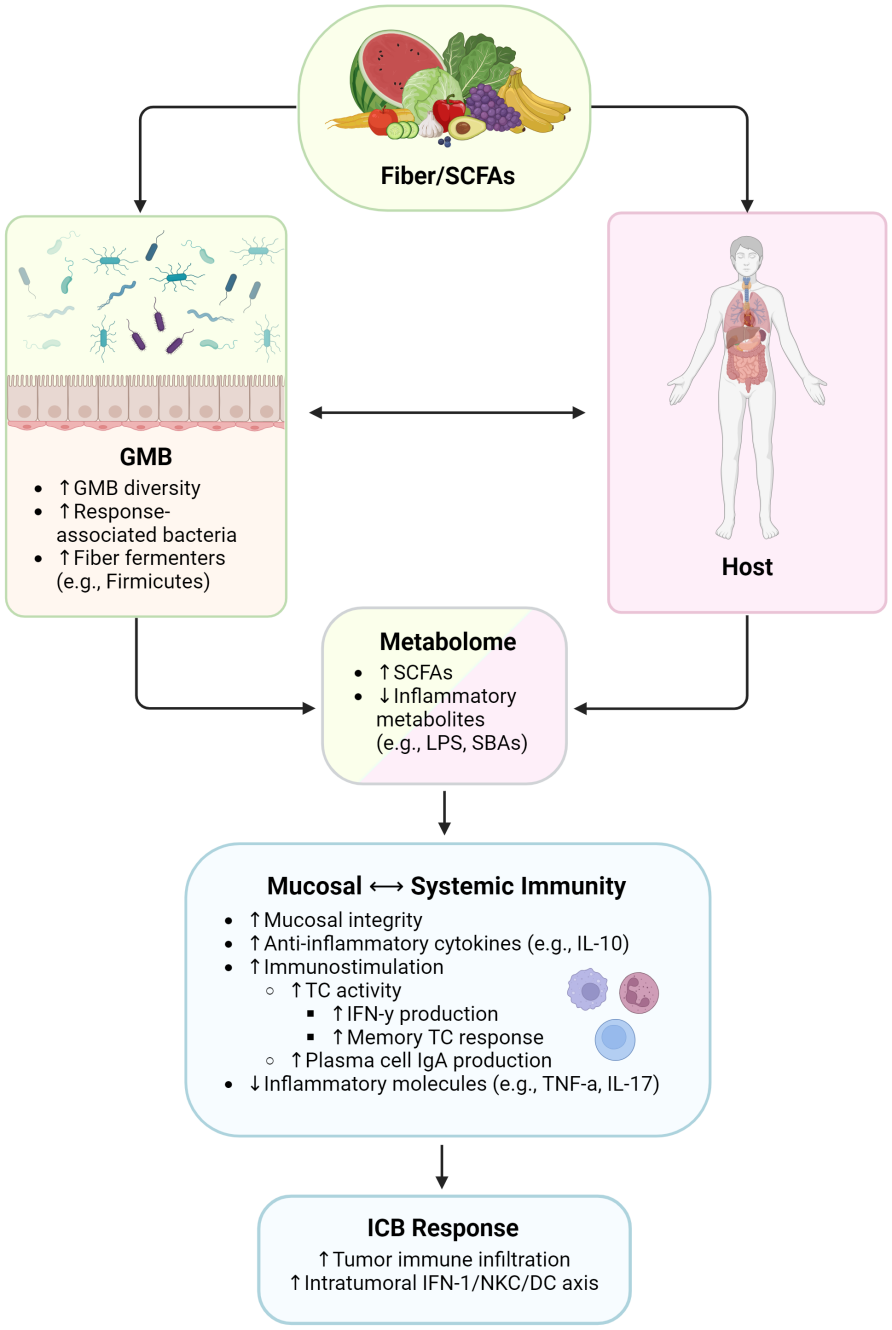
Influence of high-fiber diet and SCFAs on immunity and immunotherapy responses. SCFAs, short chain fatty acids; GMB, gut microbiome; LPS, lipopolysaccharides; SBAs, secondary bile acids; IL, interleukin; TC, T cell; IFN-γ, interferon gamma; IgA, immunoglobulin A; TNF-α, tumor necrosis factor alpha; IFN-1, type 1 interferon; NKC, natural killer cell; DC, dendritic cell. Created with BioRender.com.

**Figure 2:**
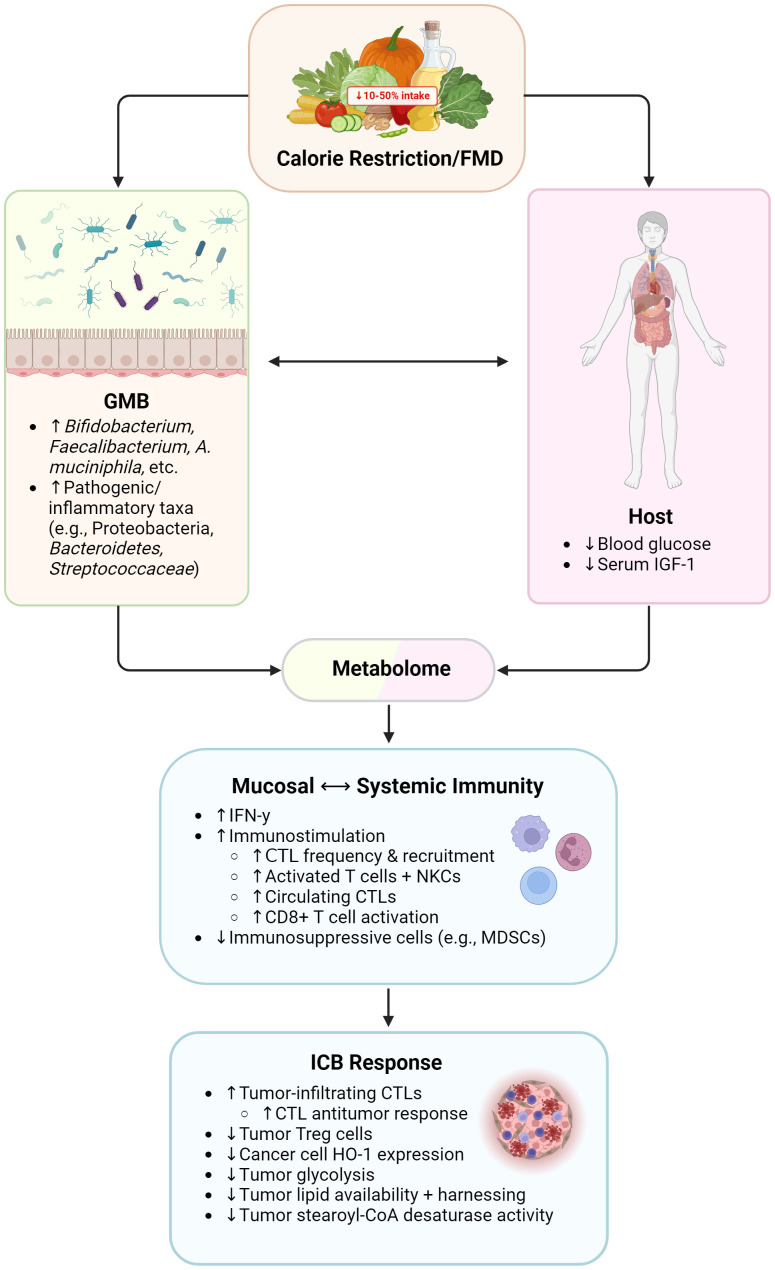
Influence of ketogenic diet on immunity and immunotherapy responses. GMB, gut microbiome. Created with BioRender.com.

**Figure 3:**
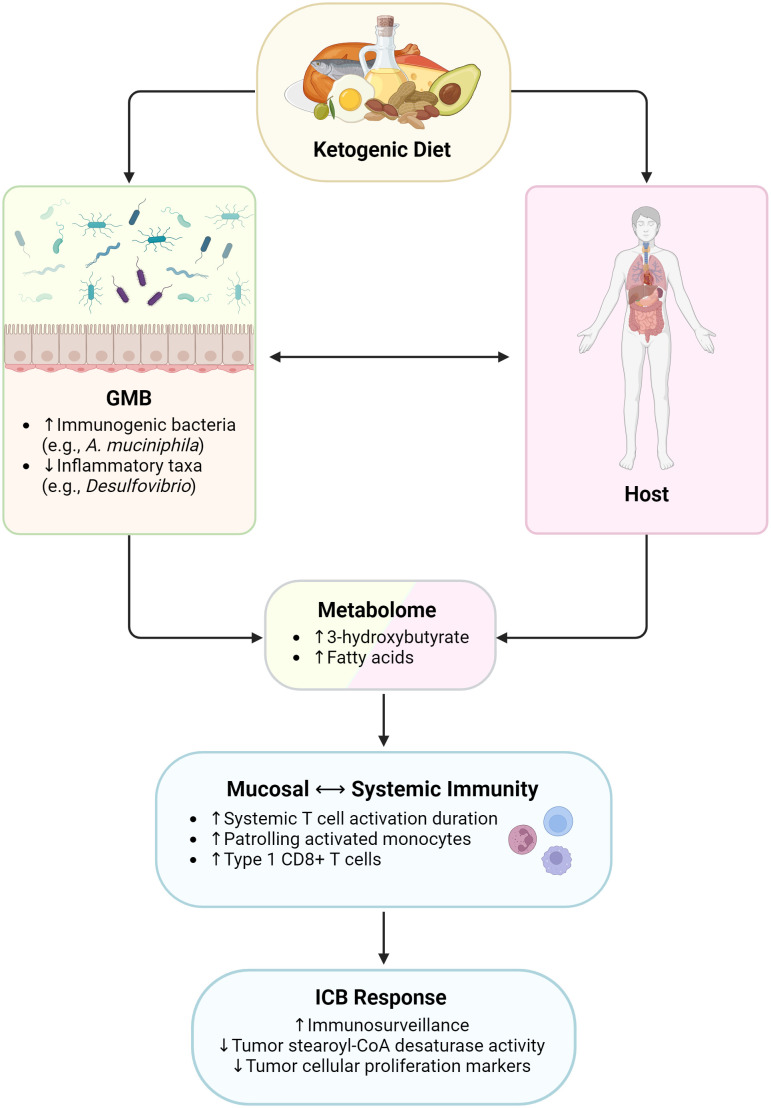
Influence of calorie restriction/fasting-mimicking diet on immunity and immunotherapy responses. FMD, fasting-mimicking diet; GMB, gut microbiome; IGF-1, insulin-like growth factor 1; IFN-γ, interferon gamma; CTL, cytotoxic T lymphocyte; NKCs, natural killer cells; MDSCs, myeloid-derived suppressor cells; Treg cells, regulatory T cells; HO-1, heme oxygenase 1. Created with BioRender.com.

In short, diet influences the GMB, which is potentially associated with clinical cancer outcomes and efficacy in ICB responses (**Figures 1**–**3**) via various possible mechanisms (**Table 1**). Although data is still emerging, based on published preclinical and human studies (**Table 2**), we recommend avoiding broad-spectrum antibiotic treatment before and during ICB and advise against therapies employing untargeted commercial probiotic supplements. We also recommend incorporating professional dietary assessments and counseling in patient consultations, and we encourage patients to consume a diverse diet rich in natural probiotics, plant species/plant-based foods, and dietary fiber. However, we advise patients to consider that more investigation is needed on dietary interventions such as KD and FMD, and that these diets should only be pursued under clinical supervision. The interactions between food components, gut microbes, the host immune system, and the genetic/immune characteristics unique to different cancers are complex and heterogeneous. Nevertheless, findings on the GMB-mediated effects of dietary interventions to improve antitumor immunity and ICB response support the pursuit of further, large-scale research to develop new interventions that could synergize with existing immunotherapy regimens to hopefully improve outcomes.

**Table 1:** Dietary pattern/ingredient effects on the GMB and possible immunomodulatory mechanisms.

Dietary Pattern/Ingredient	GMB Effects	Possible Immunomodulation Mechanisms & Immune Effects
Dietary Pattern/Ingredient (Potentially Detrimental)		
**Animal-based diet**	↑bile-tolerant microbes (151) ↑putative detrimental bacteria (152) (e.g. *Bilophila wadsworthia*) ↓putative beneficial bacteria (78) (e.g. *Roseburia* spp., *Lactobacillus* spp., *E. rectale*)	↑inflammatory metabolic products/cytokines (23, 153) (e.g. LPS, SBAs, TMAO) ↑mucosal barrier breakdown (67) ↑proteolytic fermentation (22) ↑platelet hyperresponsiveness (154) ↓SCFAs (23)
**Western-based diet (high fat, sugars, & additives)**	↓GMB diversity (155) ↑putative pathogenic taxa (35, 156) (e.g. *Proteobacteria*, LPS-producing *Enterobacteriaceae, Bilophila*) ↓putative beneficial taxa (23, 151, 157) (e.g. *Bifidobacteria*)	↑insulin resistance (158) ⇨ ↓macrophage response to pathogens (159) ⇨ ↑cancer cell IR-A expression (160) ↑dyslipidemia (161) ⇨ ↓CD8a(–) DCs (162) ⇨ ↓Th1-type immunity (162) ⇨ ↓DC migration to lymph nodes (163) ↑gut myeloperoxidase activity (164) ↑Th2 differentiation (165) ↑inflammatory cytokines (166, 167) (e.g. GM-CSF, TNF-a, IL-2, IL-1B, IL-17) ↑microbial encroachment (86, 164, 168) ↓intestinal barrier integrity (155, 169) ↓mucus thickness + release into lumen ↓Th1 differentiation (165) ↓IL-10-producing Tregs (155)
**High (saturated) fats**	↑LPS-containing microbes (23) ↑putative pathogenic bacteria (170, 171) (e.g. *Proteobacteria, B. wadsworthia*) ↓fiber-fermenting Firmicutes (e.g. *Ruminococcaceae*)	↑inflammatory metabolites/cytokines (23, 67, 172–174) (e.g. LPS, SBAs, IL-1B, TNF-a, CCL1, CCL2) ↑immunosuppressive cytokines (175) (e.g. IL-1Ra, IL-10) ↑β -catenin + PPAR-d signaling (176) ↑intestinal permeability (168, 172, 173) ↑bacterial metabolite translocation ↓tight junction proteins ↑TLR4 activation (177, 178) ↑endoplasmic reticulum stress (179) ↓SCFAs (168) ↓phagocytosing granulocytes ↓goblet cells (180, 181) ↓hematopoietic stem cells in bone marrow (182) ↓hematopoietic reconstitution potential
**High (processed) sugar**	↑putative inflammatory bacteria (156, 183) (e.g. *Enterobacteriaceae*) ↓putative beneficial taxa, especially fiber-fermenting bacteria (23, 151, 183–185)	↑inflammatory cytokines (169) ↑Th17 differentiation (186) ↑T_reg_ (187) ↓WBC phagocytosis (188, 189)
Dietary Pattern/Ingredient (Potentially Beneficial)	GMB Effects	Possible Immunomodulation Mechanisms & Immune Effects
**Plant-based diet**	↑GMB diversity (190–192) ↑putative beneficial bacteria, including fiber-fermenters/SCFA- and butyrate-producers (193–197) (e.g. Firmicutes*, Bifidobacterium*) ↓putative pathogenic taxa (102, 196) (e.g. Proteobacteria, Bacteroidetes)	↓inflammatory metabolites/cytokines (191, 196–199) ↓LPS, SBAs ↓IL-6, IL-2, TNF-a ↓triglycerides (200) ↑anti-inflammatory compounds (201, 202) (e.g. flavonoids, urolithins) ↑SCFAs (193–195, 203) (e.g. butyrate) ↑IFN-γ secretion (196) ↑IL-10, TLR2, and/or TLR4-expressing DCs (204) ↑epithelial barrier function (196) ↓fecal + intestinal pH (205) ↓colorectal proliferation (196) ↓dyslipidemia (102)
**High fiber**	↑GMB diversity (57, 83, 206) ↑putative health-promoting bacteria (194, 207) (e.g. fiber-fermenting Firmicutes, *Bifidobacterium, F. prausnitzii, A. muciniphila, Roseburia* spp.) ↓pathogenic bacteria (208, 209) (e.g. *E.coli*, *Clostridium*)	(NOTE: CONTEXT-DEPENDENT) ↓carcinogenic W*nt*-signaling pathways (22) ↑butyrate + SCFA production (194, 210) ⇨ modulation of IEC/immune cell production, development, survival, function, + recruitment (211–215) ↑mucosal barrier integrity/function (83, 85, 86, 92, 209) ↑mucus production ↑tight junction proteins ↓bacterial encroachment ↓gut-mucus depletion ↓mucosal proliferation + inflammation ↑colonocyte exfoliation + differentiation (216, 217) ↑CAZyme abundance (21) ↑TC activation + interferon response (105) ↑activated CD8+ TC memory potential (92) ↓neoplastic cell growth (105, 218–221) ↑cancer cell apoptosis + tumor angiogenesis restriction ↑TC tumor infiltration
**Dietary probiotics**	↑GMB diversity (21) ↑anti-inflammatory bacteria (116, 117) (e.g. *Akkermansia*)	↓inflammatory molecules/cytokines (21, 117) ↓serum lipid peroxidation (116) ⇨ modulation of innate immune cell response (222) ↑effector memory CD4+ TCs (21) ↑intestinal barrier function (117)
**Ketogenic diet**	↑immunogenic bacteria (123) (e.g. *A. muciniphila)* ↓putative inflammatory taxa (123) (e.g. *Desulfovibrio, Turicibacter*)	↓tumor growth/cancer progression ↓tumor cellular proliferation markers (223) ↓tumor stearoyl-CoA desaturase activity (136) ↑cICB-induced Type 1 CD8+ TC splenocytes (121) ↑patrolling activated monocyte circulation (121) ↑systemic TC activation length (121) ↑CXCR3+ TC expansion (121)
**Calorie Restriction/Fasting-Mimicking Diet**	↑putative protective/beneficial taxa (144–147) (e.g. Bacteroidaceae, *Bifidobacterium, Faecalibaculum, Actinobacteria, Lactobacillus, A. muciniphila*) ↓putative pathogenic/inflammatory taxa (144, 145, 148) (e.g. Proteobacteria, *Helicobacter, Bacteroidetes, Streptococcaceae, Desulfovibrionaceae*)	↓immunosuppressive cells (107, 137) ↓Treg presence + activation in tumors ↓MDSCs ↓tumor growth/cancer progression (136, 137, 224–226) ↑TME acidification ↑TC-mediated tumor immunogenicity + cytotoxicity ↓cancer cell HO-1 expression ↓tumor glycolysis ↓tumor lipid availability + harnessing capacity ↓tumor stearoyl-CoA desaturase activity ↓tumor MUFA/SFA ratios Alteration of immune cell maturation, activation, function, + abundance (138, 227) ↑cytotoxic CD8 TC frequency + recruitment (137) ↑activated TCs + NKCs (227) ↑common lymphoid progenitor cells–(137) ↑circulating/tumor-infiltrating CD8+ lymphocytes (137) ↑CD8+ TC → activated/memory phenotype (107) ↓blood glucose + serum IGF-1 concentration (107, 136, 227) ⇨ ↓anti-apoptotic signaling (228) ⇨ ↑intratumoral CD8/Treg ratio production (with PD-1) (227) ⇨ ↑antitumor CD8 TC response (with PD-1) (227) ↑IFN- γ (107, 227) ↑autophagy (143, 229)

↑, increased; ↓, decreased; GMB, gut microbiome; LPS, lipopolysaccharides; GMB, gut microbiome; LPS, lipopolysaccharides; SBAs, secondary bile acids; TMAO, trimethylamine N-oxide; SCFAs, short chain fatty acids; IR-A, insulin receptor isoform A; DCs, dendritic cells; GM-CSF, Granulocyte macrophage colony-stimulating factor; TNF-α, tumor necrosis factor alpha; IL, interleukin; CCL1, C-C motif chemokine ligand 1; CCL2, C-C motif chemokine ligand 2; IL-1Ra, interleukin-1 receptor antagonist; PPAR-d, Peroxisome proliferator-activated receptor-delta; TLR4, toll-like receptor 4; T_reg_, regulatory T cells; WBC, white blood cells; IFN-γ, interferon gamma; TLR2, toll-like receptor 2; IEC, intestinal epithelial cell; CAZyme, carbohydrate-active enzyme; TC, T cell; cICB, combined immune checkpoint blockade; MDSCs, myeloid-derived suppressor cells; TME, tumor microenvironment; HO-1, heme oxygenase 1; MUFA/SFA, monounsaturated fatty acids/saturated fatty acids; NKCs, natural killer cells; IGF-1, insulin-like growth factor 1; PD-1, programmed cell death protein 1.

**Table 2:** Recommendations from published clinical studies investigating the role of diet/dietary components on immunotherapy in cancer patients.

Study	Cancer	Aim	Results	Reference
Abstract by Spencer et al	Melanoma	Explore correlation between lifestyle factors (dietary patterns, probiotic use, antibiotic use), the GMB (structure, diversity), and ICB response	Probiotics may negatively impact the GMB in melanoma patients. Dietary manipulation may be able to reshape the GMB into a pro-ICB-response signature (↑whole grains, ↑fiber intake, ↑overall diet quality, ↓added sugars, ↓processed meat).	(230)
NCT05083416	Head and neck cancer	Investigate effects of randomized TRE (circadian-aligned, 14-hour nightly fast without caloric reduction) administration on GMB alteration and ICB response	TRE is a safe, feasible, and effective strategy to improve ICB response, potentially via GMB-mediated pathways (e.g., lower microbial immunosuppressive metabolites).	(231)
NCT02977052 (ongoing)	Melanoma Stage III	Study associations between baseline GMB, diet, ICB response, and immune-related adverse events	Native GMB signatures and diet shape ICB response and toxicity. GMBs dominated by Ruminococcaceae (associated with higher consumption) demonstrated greater response than Bacteroidaceae-dominated GMBs. Lower fiber and omega-3 fatty acid intake (associated with lower GMBD and SCFA production) is associated with poor ICB response.	(58)
UMIN000023303	Various (melanoma, head and neck, GI, genitourinary, etc.)	Evaluate fecal SCFA in solid cancer patients treated with anti-PD-1	Higher fecal SCFAs (associated with higher fiber intake) are associated with greater GMB diversity, improved ICB response, and longer progression-free survival.	(232)
Spencer et al., 2021	Melanoma	Assess the connection between GMB composition, dietary fiber intake, and commercial probiotic use and their influence on ICB response	Habitual higher dietary fiber consumption and avoidance of commercial probiotic use are associated with improved antitumor immunity and ICB response.	(105)
Bolte et al., 2023	Melanoma	Examine dietary patterns in association with ICB response	Mediterranean diet pattern (high intake of plant-based fiber, omega-3 fatty acids, & micronutrients) is positively associated with ICB response, progression-free survival, and less immunotoxicity.	(106)
NCT03340935	Various	Investigate safety and effects of cyclic, 5-day FMD combined with standard antineoplastic therapies	Cyclic FMD is safe, feasible, and potentially beneficial for patients receiving concomitant cancer treatments. FMD can enrich response-associated immune signatures, reducing immunosuppressive cell populations and improving immunosurveillance (greater amounts/activation of CD8+ TCs and NKCs).	(142)

GMB, gut microbiome; ICB, immune checkpoint blockade; TRE, time restricted eating; GMBD, gut microbiome diversity; SCFA, short chain fatty acids; PD-1, programmed cell death protein 1; FMD, fasting-mimicking diet; TCs, T cells; NKCs, natural killer cells.

## Author contributions

N-TAN: Conceptualization, Visualization, Writing – original draft, Writing – review & editing. YJ: Conceptualization, Supervision, Visualization, Writing – review & editing. JM: Conceptualization, Funding acquisition, Supervision, Writing – review & editing.
